# Phenotypic Characterization of Male *Tafazzin*-Knockout Mice at 3, 6, and 12 Months of Age

**DOI:** 10.3390/biomedicines11020638

**Published:** 2023-02-20

**Authors:** Michelle V. Tomczewski, John Z. Chan, Zurie E. Campbell, Douglas Strathdee, Robin E. Duncan

**Affiliations:** 1 Department of Kinesiology and Health Sciences, Faculty of Health, University of Waterloo, 200 University Ave West, BMH1044, Waterloo, ON N2L 3G1, Canada; 2 Transgenic Technology Laboratory, Cancer Research UK Beatson Institute, Switchback Road, Glasgow G61 1BD, UK

**Keywords:** tafazzin, cardiolipin, Barth syndrome, mitochondria, energy metabolism, exercise capacity, mice

## Abstract

Barth syndrome (BTHS) is an X-linked mitochondrial disease caused by mutations in the gene encoding for tafazzin (*TAZ*), a key enzyme in the remodeling of cardiolipin. Mice with a germline deficiency in *Taz* have been generated (*Taz*-KO) but not yet fully characterized. We performed physiological assessments of 3-, 6-, and 12-month-old male *Taz*-KO mice, including measures of perinatal survival, growth, lifespan, gross anatomy, whole-body energy and substrate metabolism, glucose homeostasis, and exercise capacity. *Taz*-KO mice displayed reduced viability, with lower-than-expected numbers of mice recorded at 4 weeks of age, and a shortened lifespan due to disease progression. At all ages, *Taz*-KO mice had lower body weights compared with wild-type (*Wt*) littermates despite similar absolute food intakes. This finding was attributed to reduced adiposity and diminutive organs and tissues, including heart and skeletal muscles. Although there were no differences in basal levels of locomotion between age-matched genotypes, indirect calorimetry studies showed higher energy expenditure measures and respiratory exchange ratios in *Taz*-KO mice. At the youngest age, *Taz*-KO mice had comparable glucose tolerance and insulin action to *Wt* mice, but while these measures indicated metabolic impairments in *Wt* mice with advancing age that were likely associated with increasing adiposity, *Taz*-KO mice were protected. Comparisons across the three age-cohorts revealed a significant and more severe deterioration of exercise capacity in *Taz*-KO mice than in their *Wt* littermate controls. The *Taz*-KO mouse model faithfully recapitulates important aspects of BTHS, and thus provides an important new tool to investigate pathophysiological mechanisms and potential therapies.

## 1. Introduction

The enzyme tafazzin (TAZ) is a phosphatidylcholine-monolysocardiolipin transacylase that catalyzes the remodeling of nascent cardiolipin, which is typically enriched in saturated and monounsaturated fatty acids, to form tetralinoleoyl-cardiolipin (L4-cardiolipin) [1]. TAZ is abundant in organs with high energy demands, such as the heart, where L4-cardiolipin is critical for normal bioenergetic metabolism, but this enzyme is also found in most other organs at varying levels [1]. The loss of functional TAZ leads to a deficiency in L4-cardiolipin, which causes structural and functional defects in mitochondria [2].

In humans, mutations in the *tafazzin* (*TAZ*) gene in region q28 on chromosome X cause Barth syndrome (BTHS), a rare, recessive disorder that primarily affects males [3]. The disease shows variable presentation and severity with a wide spectrum of clinical features, including cardiomyopathy, skeletal myopathy, exercise intolerance, neutropenia, growth delay, and 3-methylglutaconic aciduria [4]. The incidence of BTHS is reported to be 1.5 cases in 1,000,000 live births [5], but only 230–250 cases have been identified worldwide [6]. This discrepancy between incidence and prevalence likely stems in part from early mortality, which contributes to the significant underdiagnosis of BTHS [6]. Despite advances in symptom management, BTHS patients report a lower quality of life, and the disease still carries a substantial burden of morbidity and mortality [7,8].

The *TAZ* gene is evolutionarily conserved, and yeast, drosophila, and zebrafish have served as model organisms to study the function of TAZ and the consequences of deficiency [9,10,11,12]. Additionally, approximately 10 years ago, the first mammalian model of BTHS was generated, which employed a short-hairpin RNA-mediated knockdown of *Taz* expression (*Taz*-KD) in mice [13,14]. The *Taz*-KD mouse model has provided valuable insights into basic disease mechanisms of BTHS and has allowed testing of potential therapies [15,16,17,18,19,20]. However, a major limitation of the *Taz*-KD mouse model is the adult-onset cardiomyopathy, which is incongruous with the infantile cardiomyopathy that devastates many BTHS patients [13,21]. Thus, more recently, BTHS mouse models based on Cre-LoxP recombination have been developed, including a constitutive knockout (*Taz*-KO) [22] and a cardiomyocyte-specific knockout (*Taz*-cKO) [23,24].

Studies have begun to provide data on these newly developed Cre-mediated knockout mouse models of BTHS. The cardinal symptom, cardiomyopathy, has received priority in the study of pathophysiology, pathogenesis, and potential therapies in *Taz*-KO [22] and *Taz*-cKO mice [23,24]. Investigations into skeletal muscle and immune function have also been conducted in *Taz*-KO mice [22,25,26]. Thus, characterization efforts in *Taz*-KO mice have largely addressed only the clinical triad of BTHS, while other significant symptoms in patients such as growth delay, altered substrate metabolism and whole-body energetics, and impaired physical activity have not yet been investigated or considered in the context of aging. The phenotypic characterization of the *Taz*-KO mouse model is incomplete without consideration of these aspects of the syndrome, especially since these features have implications for the cardioskeletal pathology of BTHS across the lifespan.

The current study aimed to characterize the *Taz*-KO mouse model by investigating gross anatomy, whole-body energy and substrate metabolism, physical activity levels, systemic glucose metabolism, and maximal exercise capacity at 3, 6, and 12 months of age. We hypothesized that the *Taz*-KO mouse would be biologically congruent with the human condition of BTHS, and therefore that the characterization of its functional and metabolic characteristics at 3, 6, and 12 months of age would provide important information on disease progression across the lifespan, and would also identify useful outcome measures for preclinical trials.

## 2. Materials and Methods

### 2.1. Animals

Male *Taz*-KO mice and their wildtype (*Wt*) littermate controls were studied, since BTHS is a recessive X-linked disorder, and boys and men constitute almost the entirety of the patient population [2]. All animal procedures were performed with the approval of the University of Waterloo Animal Care Committee (AUPP#30055 (17-19), approved 25 July 2017; AUPP#41822, approved 27 August 2020; AUPP#43431, approved 9 July 2021) and comply with guidelines of the Canadian Council on Animal Care. Mice were group-housed with their littermates (at a maximum of 5 animals per cage) in a temperature- and humidity-controlled environment, on a 12:12 h light/dark cycle, with free access to standard rodent chow (Teklad 22/5 Rodent diet from Envigo, Haslett, MI, USA) and water. 

The generation of *Taz*-KO mice at the Cancer Research UK Beatson Institute and the confirmation of gene ablation have been described previously [22,27]. Experimental mice were produced by breeding heterozygous females with a germline deletion of exons 5–10 of the *Taz* gene with wild-type male C57BL/6J mice (Jackson Labs, Bar Harbor, ME, USA) to generate wildtype (*Wt*) male (*Taz*^+/Y^) and female (*Taz*^+/+^) mice, heterozygous female (*Taz*^Δ/+^) mice, and hemizygous null (*Taz*^Δ/Y^ [*Taz*-KO]) male mice. All mice used in experiments were progeny from >20 backcrosses with C57BL/6J mice.

Mice were genotyped by polymerase chain reaction with FastStart^TM^ PCR Master Mix (Roche Life Science, Mississauga, ON, Canada) using a T100 thermocycler (Bio-Rad, Mississauga, ON, Canada) under the following conditions: 95 °C for 4 min; 39 cycles of 95 °C for 30 s, 56 °C for 30 s, 72 °C for 1 min; and a final extension step of 72 °C for 7 min, using two sets of primers that contained the same reverse primer. The first set of primers, WT-U1 (5′-CTTGCCCACTGCTCACAAAC-3′) and WT-D1 (5′-CAGGCACATGGTCCTGTTTC-3′), generated a 383 bp amplicon from the *Wt Taz* allele. The second set of primers, KO-U1 (5′-CCAAGTTGCTAGCCCACAAG-3′) and WT-D1 (5′-CAGGCACATGGTCCTGTTTC-3′), generated a 280 bp amplicon when the KO *Taz* allele was present. Amplicon sizes were analyzed by electrophoresis using 1% TAE-agarose gels with ethidium bromide, and bands were visualized under ultraviolet light.

### 2.2. Genotype Ratios at 4 Weeks, Growth Rates, and Adult Survival Analysis

Mice were ear notched at the time of sexing (at 4 weeks of age) and genotyped to determine the total number of *Taz*-KO and *Wt* mice in litters at that time. A growth curve was generated by measuring weekly body weights of mice from sexing to 75 weeks of age. Thirteen litters that had at least one *Taz*-KO and one *Wt* mouse were selected to form the longevity cohort and did not undergo physiological assessments. These mice were allowed to age-out to their natural, humane lifespan endpoints, which was defined as the time when hindlimb weakness became debilitating to a degree that would soon prevent the animal from naturally accessing food and water. At this point, mice were euthanized and the age was recorded as the survival endpoint. Survival endpoint dates were also recorded for all mice that died natural deaths.

### 2.3. Indirect Calorimetry

Mice underwent indirect calorimetry using the Comprehensive Lab Animal Monitoring System (CLAMS, Columbus Instruments, Columbus, OH, USA). In preparation for CLAMS, mice were single-housed in standard cages for a 24 h period, during which time food consumption was measured. Mice were then tested in sealed, clear, individual chambers where they had free access to water and standard chow. The chambers were supplied with air at 0.5 L/min and maintained at room temperature (22–23 °C) with a 12:12 h light/dark cycle (light 07:00–19:00 h, dark 19:00–07:00 h). Immediately before the start of a CLAMS testing session, the gas sensors were calibrated with standard mixtures of gases (20.5% oxygen, 0.5% carbon dioxide, and nitrogen balance). Throughout the study, rates of oxygen consumption (VO_2_; mL/kg/h) and carbon dioxide production (VCO_2_; mL/kg/h) in each chamber were determined at 28 min intervals. The gas exchange data were used to calculate the respiratory exchange ratio (RER) as the ratio of VCO_2_ to VO_2_ and total energy expenditure (TEE) [calculated using the Lusk equation ((3.815 + 1.232 × RER) × VO_2_ (in liters))], with TEE normalized to total body weight (kg) [28]. Each chamber also used photobeams situated in rows above the cage floor to monitor total locomotor activity by infrared beam breaks along the x-(locomotion), y-(ambulation), and z-(rearing) planes. All parameters (VO_2_, VCO_2_, RER, TEE, and total locomotor activity) were measured over a total of 26 h. During the first 2 h, mice were allowed to acclimatize to the new apparatus, and these data were not included in 24 h analyses.

### 2.4. Glucose Tolerance Test (GTT)

Glucose tolerance testing (GTT) was performed essentially as we have previously described, with minor modifications [29]. Mice were fasted for 6 h (9:00–15:00 h) with access to water. At 15:00 h, baseline tail vein whole blood glucose levels were measured using the Freestyle Lite Glucose Monitoring System and test strips (Abbot Laboratories, Mississauga, ON, Canada) and then mice received an intraperitoneal (i.p.) injection of 2.0 g D-glucose (Fisher Scientific, Mississauga, ON, Canada) per kg body weight. Tail vein whole blood glucose levels were assessed at 15, 30, 60, 90, and 120 min after injection. Net incremental area-under-the-curve (iAUC) analysis was performed to determine the overall glucose excursion for each mouse [30]. 

### 2.5. Insulin Tolerance Test (ITT)

Insulin tolerance testing was performed as we have previously described with minor modifications [29]. Mice were fasted for 2 h (13:00–15:00 h) with access to water. At 15:00 h, baseline tail vein whole blood glucose levels were measured using the Freestyle Lite Glucose Monitoring System and test strips, then mice received an i.p. injection of 0.5 U insulin (Gibco, Mississauga, ON, Canada) per kg body weight. Tail vein whole blood glucose levels were assessed at 15, 30, 60, 90, and 120 min after injection. Net incremental area-over-the-curve (iAOC) analysis was performed to determine the overall glucose change for each mouse [30].

### 2.6. Treadmill Exercise Capacity Test

Exercise capacity was determined using an incremental exercise test modified from previous protocols [31,32]. All sessions were performed in the evening (16:00–19:00 h) in a dark room with overhead red illumination on a five-lane motor-driven treadmill (Model LE8700, Panlab/Harvard Apparatus, Barcelona, Spain) with a fixed slope of 5°. Days 1 to 3 were used to acclimatize the mice to the apparatus. On days 1 and 2 the mice were placed on the stationary treadmill for 5 min, and then trained to run at 5 cm/s for 5 min, 10 cm/s for 2 min, and then 15 cm/s for 3 min. On day 3 the mice spent 5 min on the static treadmill, then trained at 5 cm/s for 3 min, 10 cm/s for 2 min, 15 cm/s for 2 min, and finally 20 cm/s for 3 min. Day 4 was a rest day. On day 5, mice were placed on the stationary treadmill for 5 min, then tested for exercise capacity using an initial speed of 10 cm/s, which increased by 3 cm/s every 2 min thereafter to a maximum speed of 70 cm/s. Mice ran until reaching exhaustion, which was defined as a failure to run for 5 consecutive seconds. Running time until exhaustion and total distance ran were recorded.

### 2.7. Statistical Analyses

Comparisons between *Wt* and *Taz*-KO mice were conducted using a two-way ANOVA to detect an interaction between genotype and age as the main factors. Following identification of significant effects, Bonferroni’s multiple comparisons tests were used to identify: (1) significant differences in means within genotypes for cross-sectional age-related changes, and (2) significant differences between age-matched animals from different genotypes.

Survival of mice is presented in Kaplan–Meier survival distributions, and significance was determined by log-rank Mantel–Cox test. When a *Taz*-KO mouse reached the humane lifespan endpoint before its *Wt* littermates, the *Wt* littermates were euthanized at the same age but were entered as censored data for survival analysis, so that only natural or humane endpoint data were analyzed. The observed distribution of offspring genotypes produced from crosses of heterozygous females with *Wt* males was compared with expected Mendelian ratios by chi-squared test.

Differences in serial measurements of blood glucose levels during the GTT and ITT between *Taz*-KO and *Wt* genotypes at the same age were analyzed using a repeated-measures two-way ANOVA with genotype and time after injection as grouping factors, followed by Bonferroni’s multiple comparisons test to identify the timepoints during GTT and ITT when blood glucose levels differed between genotypes.

To assess the overall response to glucose injection, the blood glucose response during the GTT was determined by calculating the iAUC above basal glucose concentration using the trapezoidal rule for each mouse, and averaged for each age–genotype group [30,33]. To assess the overall blood glucose excursion during ITT, the iAOC under basal glucose concentration was also calculated and averaged within each age–genotype group. Groups were compared by two-way ANOVA followed by Bonferroni’s multiple comparisons tests.

Data are presented as means ± standard error of the mean (SEM). Differences were considered statistically significant when *p* < 0.05. Statistical analyses were performed and figures were generated using GraphPad Prism version 9.0.0 for Mac (GraphPad Software, San Diego, CA, USA).

## 3. Results

### 3.1. Global Taz Deletion in Mice Alters Expected Genotype Ratios and Causes Premature Mortality in Surviving Offspring

To produce male *Taz*-KO (*Taz*^Δ/Y^) mice, heterozygous females (*Taz*^Δ/+^) were crossed with *Wt* male C57BL/6J mice, since male mice lacking TAZ are sterile. This combination can produce male and female *Wt* mice, as well as male *Taz*-KO (*Taz*^Δ/Y^) mice and heterozygous females (*Taz*^Δ/+^). Seven hundred and seven pups were produced, counted, and genotyped after sexing litters at 3–4 weeks of age, and *Taz*-KO mice were found to make up only 6.1% of the total pups, which was significantly lower than the expected Mendelian ratio of 25% (Table 1). Since counting and genotyping were only performed at 4 weeks of age, this altered ratio could reflect either embryonic or perinatal lethality, or a combination of both.

**Figure 1:**
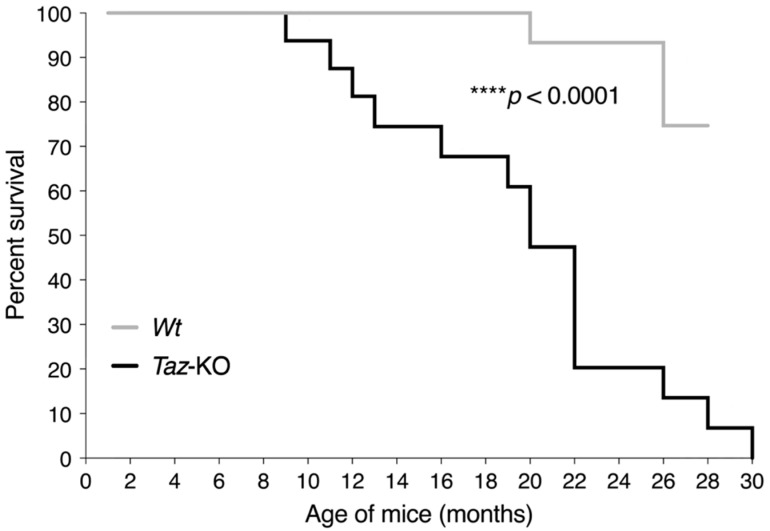
Shortened lifespan of *Taz*-KO mice. Kaplan–Meier survival curves from 1 month until 30 months of age were assessed by log-rank test for *Taz*-KO (*n* = 16) and *Wt* (*n* = 21) mice.

Among mice surviving to weaning, we observed a reduced lifespan for *Taz*-KO mice compared with their *Wt* littermates, although the earliest deaths did not occur until ~9 months of age (Figure 1). *Taz*-KO mice lived a median lifespan of 20 months, and the maximum age reached by a *Taz*-KO mouse was 30 months. In some instances, mice were found dead, in which case the definitive basis for death was unknown. However, euthanasia accounted for the majority of *Taz*-KO mouse deaths, since ethical protocols required euthanasia when mice developed hind limb weakness and poor mobility preventing unaided access to food and water.

### 3.2. Taz-KO Mice Have Lower Body Weights but Similar Food Intakes

**Figure 2:**
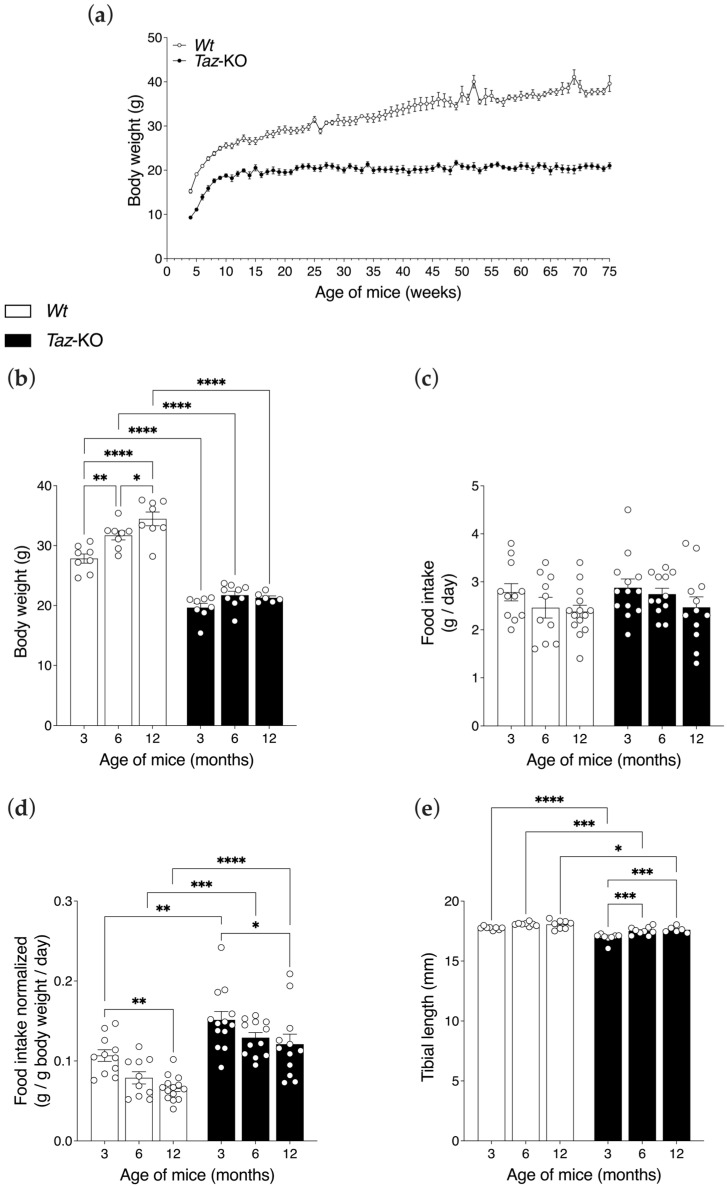
*Taz*-KO mice display a growth deficiency and smaller physical size at all ages measured. Growth curve showing the body weight of mice from 4 to 75 weeks of age (*n* = 10) (**a**). Body weight (*n* = 6–8) (**b**), absolute food intake (*n* = 10–14) (**c**), food intake normalized to total body weight (*n* = 10–14) (**d**), and tibial lengths (*n* = 6–8) (**e**), were determined at 3, 6, and 12 months of age. Data are means ± SEM. Statistical analyses were performed using two-way ANOVA followed by Bonferroni’s multiple comparisons tests. * *p* < 0.05, ** *p* < 0.01, *** *p* < 0.001, **** *p* < 0.0001.

From the onset of measurements at 4 weeks of age, *Taz*-KO mice weighed less than their *Wt* littermates, and their weights remained lower and unchanging from 12 weeks of age and onwards, while the weights of *Wt* mice increased significantly throughout the study period (Figure 2a,b). Statistical analysis of body weights at each of the three main ages studied showed that *Taz*-KO mice had significantly less mass compared with age-matched littermate control *Wt* mice at ages 3, 6, and 12 months (Figure 2b). The mean body weights of *Wt* mice increased significantly from 3 (27.8 ± 2.2 g) to 6 (31.7 ± 2.2 g) to 12 months (34.5 ± 3.2 g) of age, whereas the body weights of *Taz*-KO mice did not change significantly from 3 (19.7 ± 2.0 g) to 6 (21.7 ± 2.0 g) to 12 months (21.3 ± 0.8 g) of age (Figure 2b). This relationship resulted in a significant interaction between genotype and age (*p* < 0.05).

Surprisingly, there was no difference in absolute food consumption between *Taz*-KO and *Wt* mice that could account for the leaner phenotype, with unadjusted food consumption remaining consistent across aging in both genotypes (Figure 2c). Indeed, when adjusted for body weight, *Taz*-KO mice had significantly higher food intakes than their *Wt* littermates at all age-points, potentially indicating lower food efficiency (Figure 2d). Both *Taz*-KO and *Wt* mice also had significantly lower weight-normalized food intakes at 12 months of age compared with genotype-matched mice at 3 months of age (Figure 2d).

In contrast to differences in body weights, differences in tibial lengths were less pronounced. *Taz*-KO mice had significantly shorter tibial lengths compared with age-matched *Wt* mice at all ages assessed, although this reduction in length was minor, with only a 4% difference evident in 3-month-old mice (*Taz*-KO: 17.0 ± 0.38 vs. *Wt*: 17.8 ± 0.14 mm), a 3% difference evident in 6-month-old-mice (*Taz*-KO: 17.5 ± 0.32 vs. *Wt*: 18.1 ± 0.15 mm), and a 2% difference evident between genotypes in 12-month-old mice (*Taz*-KO: 17.6 ± 0.25 mm vs. *Wt*: 18.1 ± 0.37 mm) (Figure 2e). Thus, slightly smaller stature could not account for the drastically lower body weights observed in *Taz*-KO mice. 

Interestingly, the data did support delayed growth in *Taz*-KO mice. While tibial lengths in *Wt* mice did not change significantly between the ages measured, in the *Taz*-KO mice, tibial lengths increased significantly with age, such that 6- and 12-month-old mice had longer tibial bones than 3-month-old mice (Figure 2e). There was no difference between *Taz*-KO mice aged 6 and 12 months, indicating that growth was completed by 6 months of age in these animals. To determine the source of the significant differences in body weights, organ and tissue masses were measured.

### 3.3. Taz-KO Mice Have Diminutive Organs and Tissues and Reduced Adiposity

**Figure 3:**
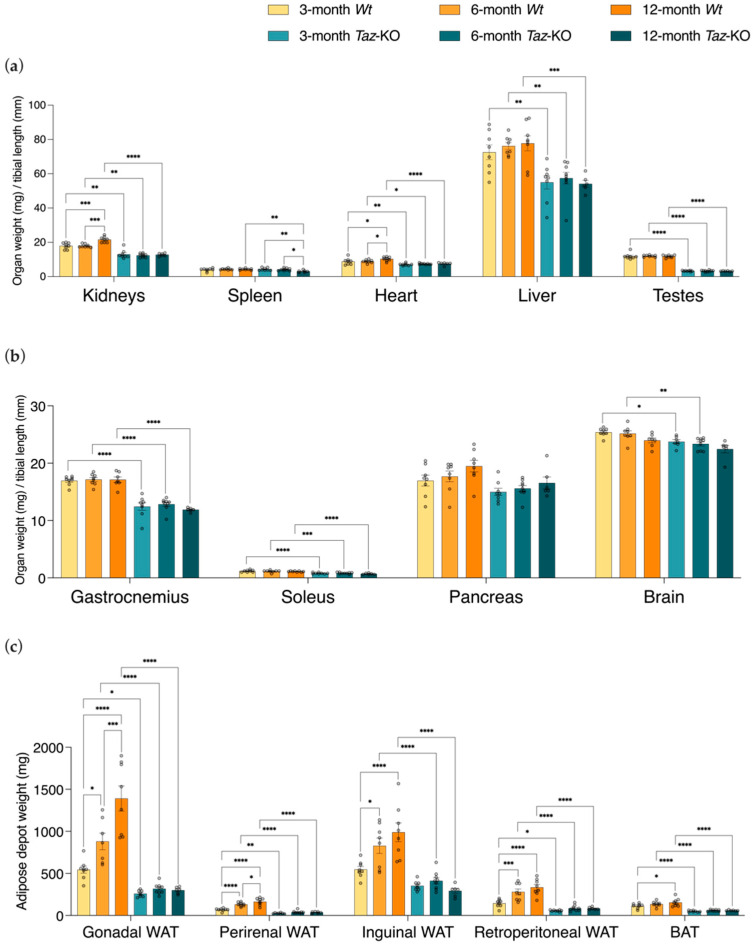
Smaller tissue weights underlie the lower body weights of *Taz*-KO mice. *Taz*-KO mice and *Wt* littermates were necropsied at 3, 6, and 12 months of age. Tibia-normalized weights of kidneys, spleen, heart, liver, and testes (**a**), gastrocnemius, soleus, pancreas, and brain (**b**) are shown. Absolute weights of gonadal, perirenal, inguinal, and retroperitoneal white adipose tissue (WAT) and brown adipose tissue (BAT) (**c**). Data are means ± SEM (*n* = 6–8). Statistical analysis was performed using two-way ANOVA followed by Bonferroni’s multiple comparisons tests. * *p* < 0.05, ** *p* < 0.01, *** *p* < 0.001, **** *p* < 0.0001.

*Taz*-KO mice had lower organ weights, skeletal muscle weights, and adipose depot weights compared with age-matched *Wt* control mice in nearly all comparisons performed (Figure 3a–c). Due to the drastic differences in body weights, organ and tissue masses were normalized to tibial lengths rather than body masses [33]. Age-matched *Wt* mice had larger kidney weights compared with *Taz*-KO mice at all three ages examined (Figure 3a). Kidney masses were significantly elevated in 12-month-old *Wt* mice compared with either 3- or 6-month-old *Wt* mice, but remained stable in weight across all three ages in *Taz*-KO mice (Figure 3a). This relationship resulted in a significant interaction between genotype and age for kidney organ weights (*p* < 0.01).

There was also a significant genotype-by-age interaction for spleen weights (*p* < 0.05). The spleens of *Wt* mice did not change in weight between the different ages analyzed (Figure 3a). Conversely, spleens of *Taz*-KO mice were significantly smaller at 12 months of age than at 3 and 6 months of age. This appears to have contributed to findings that 12-month-old *Taz*-KO mice also had smaller spleens than their age-matched *Wt* littermates, whereas no significant difference was evident between genotypes when younger mice were compared.

*Taz*-KO mice had lower heart weights than age-matched *Wt* control mice at all three ages (Figure 3a). Heart weights of *Wt* mice were significantly greater at 12 months of age than at either 3 or 6 months of age, but heart weights of *Taz*-KO mice did not differ significantly across the ages examined. However, there was no significant genotype-by-age interaction effect for heart weights. 

The liver and testes did not change in weight with age in either genotype, and both organs were significantly smaller in *Taz*-KO mice than their age-matched control littermates at each age measured (Figure 3a). This was particularly pronounced in the case of the testes, which in *Taz*-KO mice were less than one-third the mass of age-matched *Wt* mice. 

The weights of gastrocnemius and soleus skeletal muscles were significantly smaller (~25% lower at 3 months of age) in *Taz*-KO mice compared with their age-matched *Wt* littermate mice, but did not change significantly with age in either genotype (Figure 3b).

Interestingly, no differences in mean pancreas weights were observed between *Taz*-KO mice and *Wt* littermates at any age-point (Figure 3b). Although brain masses were significantly affected, the differences were small (Figure 3b). Brains of *Taz*-KO mice were 6% smaller than *Wt* littermate control mice at 3 months of age, and 7% smaller at 6 months of age, but the significant difference between genotypes disappeared by 12 months of age. Overall, the tissues and organs examined appeared grossly morphologically normal despite the smaller size.

A striking difference in adiposity was apparent between the two genotypes, and the absolute masses of all measured white adipose tissue (WAT) depots were significantly smaller in *Taz*-KO mice at all age-points, with the exception of inguinal WAT, which was not yet different between genotypes at 3 months of age (Figure 3c). For example, the gonadal and perirenal WAT depots were 52% and 64% smaller, respectively, in 3-month-old *Taz*-KO mice in comparison with their age-matched *Wt* littermates. Gonadal, perirenal, inguinal, and retroperitoneal WAT depots in *Wt* mice increased in weight as the mice aged, while there was no significant change in the masses of these adipose depots from the *Taz*-KO mice with age, and as such there was a significant genotype-by-age interaction effect for all measured WAT depots (gonadal WAT and perirenal WAT: *p* < 0.0001; inguinal WAT and retroperitoneal WAT: *p* < 0.01). Brown adipose tissue also significantly increased in weight with age in *Wt* mice, increasing from ages 3 to 12 months. However, brown adipose tissue masses remained unchanged across these ages in *Taz*-KO mice, and were significantly smaller in *Taz*-KO mice compared with age-matched *Wt* littermates at each age-point measured.

### 3.4. Ambulatory, Locomotor, and Rearing Activity in Taz-KO and Wt Littermates

**Figure 4:**
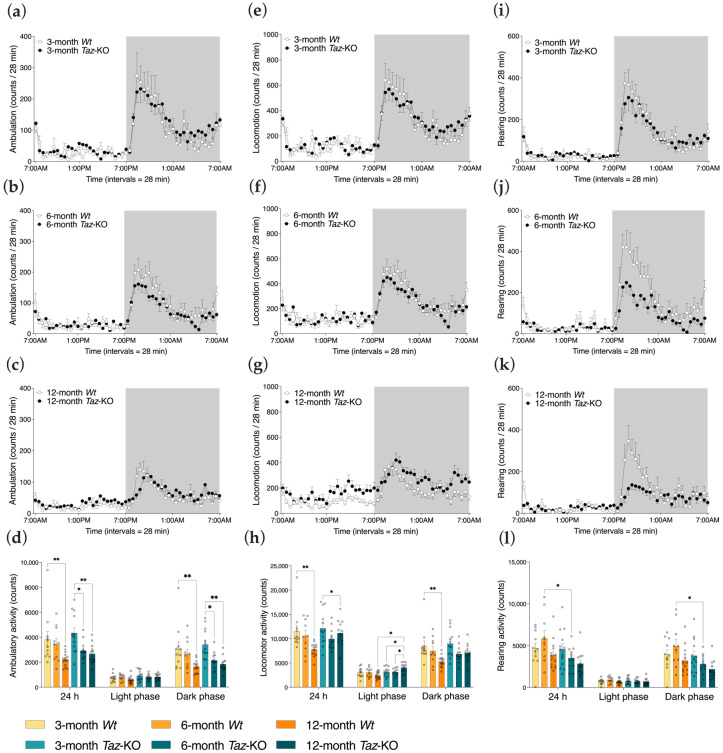
Basal activity levels are largely comparable in age-matched *Taz*-KO and *Wt* mice. Three separate mouse cohorts, aged 3, 6, and 12 months, were housed in metabolic chambers equipped with infrared beams. Representative plot of sums of infrared beam breaks per 28 min interval caused by ambulation movements, such as when a mouse transverses a chamber (**a**–**c**), locomotion movements, such as grooming and feeding in addition to ambulation (**e**–**g**), and rearing movements (**i**–**k**) over a period of 24 h. Dark (denoted by shaded background) and light (denoted by unshaded background) cycles are shown. The corresponding bar graphs of the total sum, in addition to sums per photoperiod, of beam breaks caused by ambulatory activity (**d**), locomotor activity (**h**), and rearing activity (**l**) for the cohorts. Statistical analysis was performed using two-way ANOVA followed by Bonferroni’s multiple comparisons tests (**d**,**h**,**l**). Data are means ± SEM (*n* = 10–14). * *p* < 0.05, ** *p* < 0.01.

Since the low body weights and reduced adiposity of *Taz*-KO mice occurred without a concomitant reduction in food intake, other mechanisms of energy balance offset were investigated. The CLAMS system records the three-dimensional movement of mice in metabolic chambers and thus provides measures of routine activity levels. Remarkably, there were no significant differences between genotypes at any age-point in ambulatory activity, which is the activity recorded when a mouse traverses the chamber (Figure 4a–d). Both genotypes experienced age-related declines in ambulatory activity, with a significant decrease from 3 to 12 months of age for *Wt* mice, but with this decrease occurring earlier in *Taz*-KO mice, by 6 months of age (Figure 4d).

Total locomotor activity, which is the sum of all horizontal and vertical cage movements of mice, was only different between the genotypes at 12 months of age (Figure 4e–h). The total locomotor activity of *Wt* mice decreased as they aged from 3 to 12 months, with longitudinal differences most discernable during the dark phase. Specifically, in the light phase, two-way ANOVA indicated significant effects of genotype (*p* < 0.01) on total locomotor activity, with the effect of genotype dependent on age (*p* < 0.01). Conversely, *Taz*-KO mice demonstrated significantly increased total locomotor activity from either 3 or 6 to 12 months of age during the light period (Figure 4h). The light phase corresponds to the inactive phase of mice, and therefore this finding suggests possible greater disruptions in sleep cycles with aging in *Taz*-KO mice compared with *Wt* mice. 

In the z-dimension that measured rearing activity, 6-month-old *Wt* mice were significantly more active than their *Taz*-KO counterparts, but there were no differences between genotypes at 3 or 12 months of age, and overall no significant age-related declines (Figure 4i–l). 

Overall, activity measures were surprisingly similar between *Taz*-KO mice and their age-matched *Wt* littermates (Figure 4a–l). Given the cardioskeletal myopathy of the *Taz*-KO mouse model [9], it was anticipated that generally reduced activity measures would be seen. However, aside from lower 24 h and dark phase rearing activity in 6-month-old animals, there were no other significant deficits in *Taz*-KO mice relative to their age-matched controls. On the other hand, aside from elevations in 24 h and light phase locomotor activity in 12-month-olds, significant elevations in activity were also largely absent in *Taz*-KO mice, indicating that hyperactivity was not a major contributor to the lean phenotype of these animals.

### 3.5. Taz-KO Mice Have Alterations in Respiratory Gas Exchange

**Figure 5:**
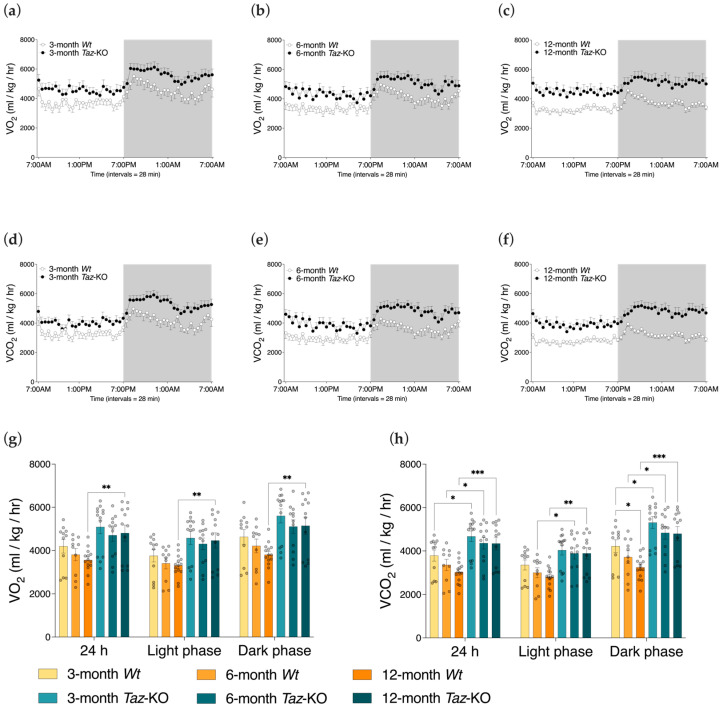
Oxygen consumption and carbon dioxide production by *Taz*-KO and *Wt* mice. Three separate mouse cohorts, aged 3, 6, and 12 months, were housed in metabolic chambers for 24 h. Representative plot of whole-body oxygen consumption rates (VO_2_) (**a**–**c**) and carbon dioxide production rates (VCO_2_) (**d**–**f**) normalized to total body weight. Dark (denoted by shaded background) and light (denoted by unshaded background) cycles are shown. The bar graphs of the daily averages, in addition to averages by photoperiod, of VO_2_ (**g**) and VCO_2_ (**h**) are shown. Statistical analysis was performed using two-way ANOVA followed by Bonferroni’s multiple comparisons tests. Data are means ± SEM (*n* = 10–14). * *p* < 0.05, ** *p* < 0.01, *** *p* < 0.001.

Compared with *Wt* littermates, *Taz*-KO mice had higher rates of oxygen consumption (VO_2_) only at 12 months of age (Figure 5a–c,g). This difference was noted in each of the light and dark cycles, and also when 24 h data were averaged. However, higher rates of carbon dioxide production (VCO_2_) by *Taz*-KO mice were noted at all three ages during the light and dark phases, and in 24 h averages, with the exception of measures taken during the light phase in 3-month-old mice (Figure 5d–f,h).

### 3.6. Taz-KO Mice Have Alterations in Total Energy Expenditure and Respiratory Exchange Ratios

**Figure 6:**
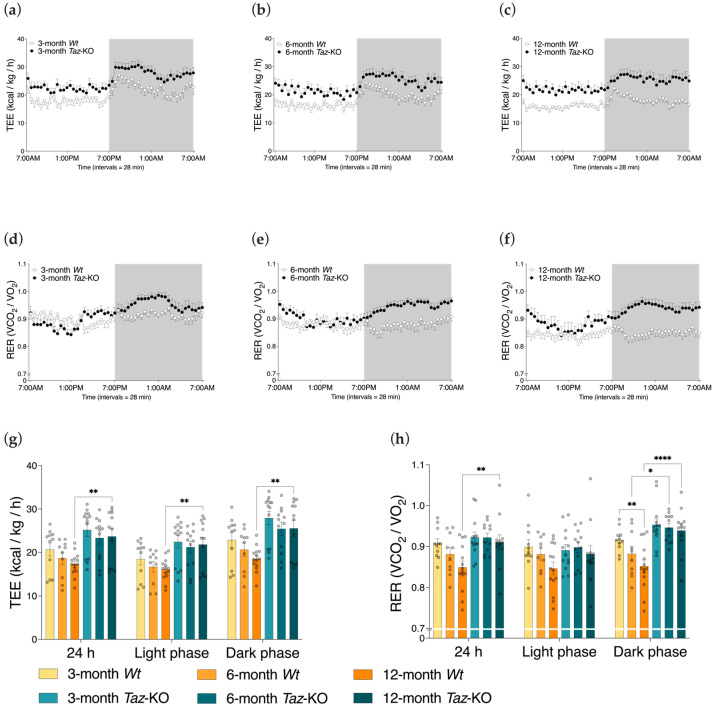
Higher energy expenditure and respiratory exchange ratios in *Taz*-KO mice. Three separate mouse cohorts, aged 3, 6, and 12 months, were housed in metabolic chambers for 24 h. Plots of mean total energy expenditure (TEE) normalized to total body weight (**a**–**c**) and respiratory exchange ratios (RER) (**d**–**f**) are shown. Dark phase (denoted by shaded background) and light phase (denoted by unshaded background) cycles are shown. Bar graphs of daily averages, in addition to averages by photoperiod, of TEE (**g**) and RER (**h**) are shown. Statistical analysis was performed using two-way ANOVA followed by Bonferroni’s multiple comparisons tests. Data are means ± SEM (*n* = 10–14). * *p* < 0.05, ** *p* < 0.01, **** *p* < 0.0001.

Total energy expenditure (TEE) was estimated through gas exchange data collected in the metabolic chambers. TEE was only different between the genotypes at 12 months of age, where it was found to be higher in the *Taz*-KO mice (Figure 6a–c,g). Within genotypes, there was no significant effect of age.

The respiratory exchange ratio (RER), indicative of the relative proportion of energy production derived from the oxidation of carbohydrates versus fatty acids, was higher during the dark phase in *Taz*-KO mice compared with their age-matched control littermates at 6 and 12 months of age (Figure 6d–f,h). Since mice are nocturnal, the dark phase was when most feeding activity occurred. These findings suggest that at these ages *Taz*-KO mice use a greater proportion of carbohydrates, and a lower proportion of fats as fuel sources in the dark cycle, in contrast to *Wt* mice. Differences in genotypes were most pronounced at 12 months of age, since 24 h RER values were also higher for *Taz*-KO mice at this age (*Taz*-KO mice: 0.94 ± 0.06 vs. *Wt* mice: 0.85 ± 0.06, *p* < 0.01). 

### 3.7. Glucose Homeostasis Is Preserved with Aging in Taz-KO but Not Wt Mice 

**Figure 7:**
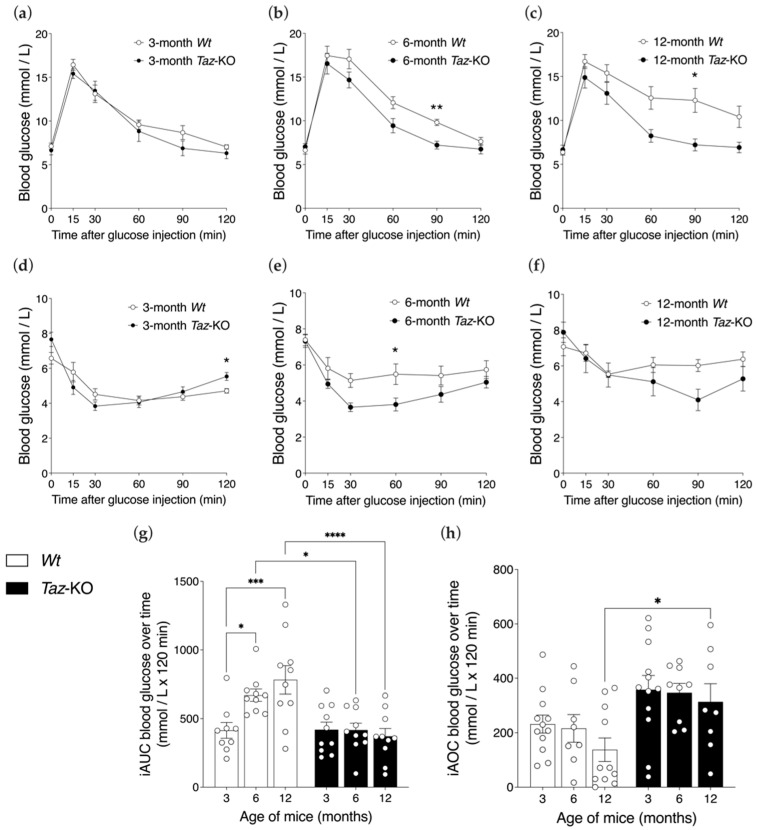
*Taz*-KO mice are protected from age-related impairments in glucose control. Glucose tolerance testing (GTT) (**a**–**c**) and insulin tolerance testing (ITT) (**d**–**f**) were performed in three age cohorts of mice. Significant differences in glycemia across timepoints during GTT or ITT were assessed by repeated-measures two-way ANOVA followed by Bonferroni’s multiple comparisons tests. * *p* < 0.05, ** *p* < 0.01. Corresponding average incremental area-under-the-curve (iAUC) for GTT (**g**) and incremental area-over-the-curve (iAOC) for ITT (**h**) are shown. Statistical analysis was performed using two-way ANOVA followed by Bonferroni’s multiple comparisons tests. * *p* < 0.05, *** *p* < 0.001, **** *p* < 0.0001. Data are means ± SEM (*n* = 9–12).

Baseline (i.e., 6 h fasted) blood glucose measures were similar between age-matched *Taz*-KO and *Wt* mice (Figure 7a–c). Glucose tolerance following i.p. glucose injection was also largely equivalent between 3-month-old *Taz*-KO and *Wt* mice (Figure 7a,g). However, differences between the genotypes became apparent with age, largely due to a decline in glucose tolerance in *Wt* mice that was not seen in *Taz*-KO mice. Overall glucose clearance in response to a glucose challenge was reduced in both 6- or 12-month-old *Wt* mice relative to 3-month-old *Wt* mice, as evidenced by larger iAUC excursions (Figure 7g). In contrast, the glucose tolerance of *Taz*-KO mice remained stable with age. Ultimately, the divergence of glucose clearance trends with age between *Wt* and *Taz*-KO mice yielded a significant genotype-by-age interaction (*p* < 0.01) and significant discrepancies in glucose homeostasis, in which 6- and 12-month-old *Wt* mice were significantly less glucose tolerant compared with age-matched *Taz*-KO mice.

Insulin tolerance testing (ITT) indicated that insulin action slightly, but non-significantly, declined in the *Wt* mice with advancing age, while the *Taz*-KO mice were protected against major age-related changes, so that at 12 months of age, *Wt* mice were insulin resistant relative to *Taz*-KO mice (Figure 7d–f,h). 

### 3.8. Aging Exacerbates the Exercise Intolerance of Taz-KO Mice

**Figure 8:**
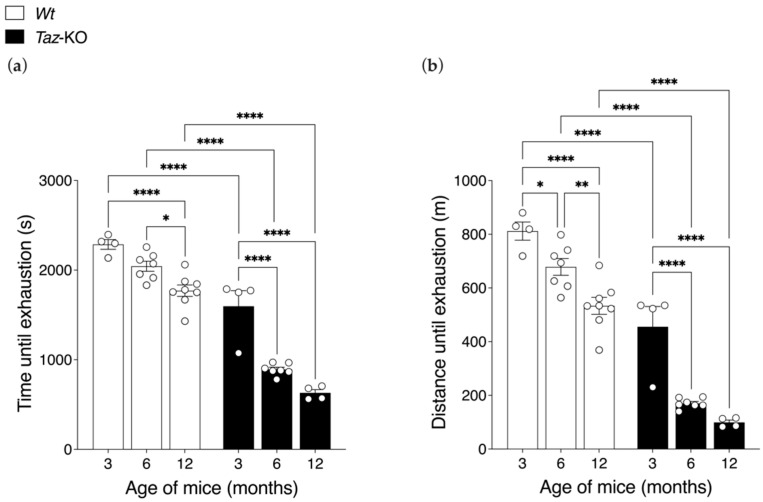
Declining exercise capacity in *Taz*-KO mice with age. Three separate age cohorts of mice ran on the treadmill until exhaustion, at which point the maximal running time (**a**) and distance ran (**b**) were recorded. Data are means ± SEM (*n* = 4–8). Statistical analysis was performed using two-way ANOVA followed by Bonferroni’s multiple comparisons tests. * *p* < 0.05, ** *p* < 0.01, **** *p* < 0.0001.

Maximal exercise capacity testing required mice to run to exhaustion on a motorized treadmill apparatus with increasing speed. *Taz*-KO mice exhibited a markedly reduced capacity to sustain running exercise, exhausting at an earlier timepoint and achieving a shorter total distance traversed compared with *Wt* littermates at all age-points (Figure 8a,b). For example, at 3 months of age, when the differences between genotypes were the smallest, *Taz*-KO mice ran an average of 26 min 36 s until exhaustion, which was 30% less time than age-matched *Wt* littermates that lasted 38 min 7 s on the treadmill (Figure 8a). Similarly, *Taz*-KO mice ran an average distance of 456 m in this test, while their *Wt* littermate controls ran an average of 812 m. 

*Taz*-KO mice also demonstrated greater- and earlier-onset declines in exercise capacity during aging compared with *Wt* littermates. In *Wt* mice, a significant decline in running-time-to-exhaustion was only evident once they reached 12 months of age, whereas *Taz*-KO mice exhibited a significant 44% decrease in running-time-to-exhaustion from 3 to 6 months of age. While both *Taz*-KO and *Wt* mice exhibited age-related declines in exercise capacity, there was a significant interaction between genotype and age (*p* < 0.01) for running-time-to exhaustion due to the severe loss of exercise capacity of *Taz*-KO mice with advancing age. When the total distance achieved was analyzed, both *Wt* and *Taz*-KO mice had significant declines from 3 to 6 months of age (Figure 8b). However, the magnitude of the decrease was only 16% in *Wt* mice, whereas in *Taz*-KO mice it was 63%. Thus, while *Wt* mice experienced a further statistically significant decline in distance achieved during the treadmill test from 6 to 12 months of age, *Taz*-KO mice, which were already substantially impaired at 6 months, did not. 

## 4. Discussion

In the current work, we characterized a new mouse model of BTHS, examining the phenotype of the *Taz*-KO mouse model at three ages corresponding to the human periods of young adulthood (i.e., 3 months), early middle-age (i.e., 6 months) and late middle-age (i.e., 12 months) [34]. We undertook this work in an effort to provide detailed data on the nature of the model and to better understand how closely it recapitulates certain aspects of the human disease. Since the oldest known individuals with BTHS are now entering late middle-age, we also anticipate that this information will help to inform a clinical understanding of the natural progression of the disease. Additionally, these data are expected to help inform decisions on the adoption of this model by researchers, particularly those engaged in preclinical studies of novel therapeutics, where a close alignment of the model system with the clinical disease is critical for accurate evaluation of efficacy.

In humans, BTHS is recognized as a cause of male fetal death resulting in miscarriage and stillbirth, with cardiac failure ascertained as a contributing factor to both fetal and neonatal deaths [35]. BTHS is also a cause of premature mortality in individuals surviving to adulthood [36]. In the present work, global *Taz* deletion negatively affected the viability of *Taz*-KO mice across their lifespan. Only 6% of mice at 4 weeks of age were *Taz*-KO (i.e., *Taz*^Δ/Y^), in contrast to the predicted proportion of 25%, indicating early life losses. Surviving mice also had a shortened lifespan due to either progressive hindlimb weakness requiring humane euthanasia, or earlier spontaneous death, which resulted in a median age of mortality of 20 months. 

In the *Taz*-KD mouse model, a causal role for developmental cardiomyopathy in pre- and perinatal lethality has been demonstrated. Induction of *Taz* knockdown leads to cardiac noncompaction and fetal loss, but only when induced at early stages (i.e., E7.5 and E10.5), and not later stages of gestation [37]. These findings strongly suggest a critical window where *Taz*-dependent mitochondrial function is required for optimal cardiogenesis and embryonic survival. However, this concept has recently been challenged by studies on *Taz*-KO and *Taz*-cKO mice [22,23]. 

Our finding of a low *Taz*-KO genotype ratio at 4 weeks of age corroborates the low birthrate and rapid perinatal lethality reported by Wang et al. (2020) in this model [22]. In that study, *Taz*-KO mice were born at a rate of ~17% at post-natal day zero, which was still below the expected Mendelian ratio of 25%; however, most liveborn *Taz*-KO mice died in the neonatal period so that the survival diminished to ~7% based on the total number of mice alive at 4 weeks of age, essentially matching the survival rate at this age that was observed in the present study. Surprisingly, however, the low neonatal survival rate was not replicated in *Taz*-cKO mice, which were born at the expected Mendelian ratio (25%), with fewer than 5% of *Taz*-cKO mice displaying perinatal lethality [23]. As a result, it has been inferred that survival of *Taz*-KO fetuses and pups is not impaired by cardiac insufficiency, per se, but rather may be explained by skeletal muscle defects [22]. While infants with BTHS have been reported to be hypotonic, lethargic, and to have feeding issues [2], medical and dietary interventions prevent mortality from these complications in human patients [38]. Thus, the recent insights from *Taz*-KO and *Taz*-cKO models suggests that research on understudied aspects of BTHS, including pathological changes in skeletal muscle, should be performed to better understand the source of embryonic and neonatal lethality in this disease.

As with the early neonatal lethality that was observed in *Taz*-KO mice, the absence of late-stage intervention distinguishes the animal model from the course of human disease. Skeletal muscle weakness became a determining factor for survival in adult *Taz*-KO mice in the present study, since they developed progressive hindlimb weakness that required humane euthanasia, or died a natural death due to unknown causes, at a median age of 20 months, while *Wt* mice live to a median age of 27 to 29 months according to this and other studies [39]. It is likely that the natural lifespan of *Taz*-KO mice would be longer if the mice were given assistance. While progressive skeletal myopathy has not been formally studied in humans due to a lack of advanced-age patients, interviews with individuals who have BTHS have revealed personal accounts of worsening muscle weakness and fatigue with aging [7]. Comparison of skeletomuscular parameters in pediatric versus adult BTHS patients have indicated progressive muscle weakness, further highlighting the need for research on the skeletal phenotype of this disease [40].

Notably, as mice that developed to maturity did not begin to die spontaneously until middle-age (~9 months), this tentatively implies that cardiac function is relatively well-preserved in surviving *Taz*-KO mice, as was also seen in *Taz*-cKO mice up to 1 year of age [23], and parallels the human condition [5,41,42]. Longitudinal clinical studies on BTHS patients reveal that cardiac function is heterogenous across the BTHS population and variable over time in an individual patient, but generalize that cardiac function tends to stabilize and is only mildly abnormal in most subjects beyond infancy, although most patients are maintained on indefinite medical therapy for heart failure [5,41,42]. However, it is interesting to note that heart weight did not significantly change with age in our global *Taz*-KO mice, and therefore does not undergo overt hypertrophic cardiac growth, offering further evidence that cardiac function, although likely compromised, is also likely stable in mature animals in this model.

The most prominent aspect of the adult global *Taz*-KO mouse phenotype that we observed was the dramatically lower body weight. Interestingly, tibial lengths were only slightly smaller, by ~4% in *Taz*-KO mice at 3 months of age, revealing that these mice had skeletal frame sizes that were largely comparable to *Wt* littermates. Rather, differences in lean and adipose tissue masses, coupled with diminutive organ sizes, were responsible for the significantly reduced body masses. Mean weights of gastrocnemius and soleus skeletal muscles were dramatically lower, by ~30%, as were masses of major organs such as heart, liver, and kidneys, compared with measures in age-matched littermate controls. In this regard, BTHS patients also reach normal adult heights following a delayed post-pubertal growth spurt [41], but have lower percent and absolute skeletal muscle masses, as indexed by fat-free mass measurements in body composition assessments [43,44].

The only organ that significantly changed in mass with aging in the *Taz*-KO mice was the spleen, which became progressively smaller from 3 and 6 to 12 months of age. The spleen is critical for immune system function, since it is the key site for T cell activation and B cell differentiation into plasma cells, which are the primary effector cells of the adaptive immune system [45]. This change in spleen weight may have relevance for understanding the clinical pathology of BTHS, since atrophy of the spleen is expected to result in impaired immunity [46]. *Taz*-KD and *Taz*-KO mice show immunodeficient phenotypes with impaired B cell [47] and T cell [25] function, respectively. Although neutropenia is a hallmark of BTHS, defects in other immune cell types may also be involved in the pathogenesis of infections in this disease, and warrant further investigation since severe infections are the second most common cause of hospitalization in this patient population [41].

In contrast to the sizeable effects in most organs, brain masses were relatively well conserved in *Taz*-KO mice at all ages. This aligns well with the presence of only minimal central nervous system involvement in BTHS [48], and is a deviation from other inherited mitochondrial disorders where the nervous system, which has a high energetic demand, is typically compromised [49]. This can be postulated to be due to the greater cardiolipin heterogeneity found in the brain [50] and, hence, lower dependency of brain mitochondria on TAZ-mediated production of L4-cardiolipin. Notably, reports of cognitive difficulties among patients do exist, including lower visual–spatial skills and impaired mathematical ability [51], and *Taz*-KD mice studies have raised concerns of anxiety and memory issues [52]. The generation of brain-specific KO models of *Taz* would help to better understand the role of this enzyme in the central nervous system. 

In addition to reductions in lean body organs and tissues, 40–60% reductions in adipose tissue depot masses were recorded in *Taz*-KO mice compared with their age-matched *Wt* littermates at 3 months of age. This disparity increased progressively, since the *Taz*-KO mice did not exhibit the age-related fat mass accumulation that was characteristic of the *Wt* mice. This aspect of the mouse phenotype was in contrast to that observed in BTHS patients, who tend to have greater absolute fat masses and body fat percentages compared with age- and height-matched controls [44]. Notably, however, additional factors related to disease management may influence the human phenotype. In particular, the common use of beta-blockers in this clinical population [44], which inhibit adipose tissue lipolysis, and therefore can increase fat mass preservation [53], may be a factor. Regardless, the dramatic difference in adiposity between *Taz*-KO and *Wt* littermates, as well as the resistance these mice showed to age-related fattening, suggests that further studies on the role of TAZ in adipose tissue may yield findings relevant to disorders affected by obesity. 

Body composition, and particularly adiposity, is intricately tied to a balance between energy intake and energy expenditure. Despite their smaller size, *Taz*-KO mice consumed the same absolute amount of food as their *Wt* littermates, and could even be considered hyperphagic compared with *Wt* mice when chow intakes were normalized to body weights. Although intestinal absorption was not measured to ensure digestive sufficiency, a previous study found no impairments in nutrient absorption in *Taz*-KD mice [54], which implies that the source of the lean phenotype of *Taz*-KO mice lies in the other major component of the energy balance equation, energy expenditure.

We initially hypothesized that *Taz*-KO mice would display lower levels of basal locomotor activity, and therefore increased physical activity would not account for the lean phenotype. We were surprised, however, to find that *Taz*-KO mice had levels of physical activity that were generally similar to their age-matched *Wt* littermates. Skeletal myopathy is a prominent symptom of BTHS, and patients commonly report chronic fatigue, exercise intolerance, pain, postural imbalance, delayed motion reaction time, and cognitive difficulties [7,55,56,57] that result in lower levels of daily activity [40]. It is unknown why the *Taz*-KO mice did not exhibit reduced daily activity, but this finding agrees with *Taz*-KD mouse studies, which also report either no differences or higher levels of physical activity versus controls [17,58]. One possible explanation is that spontaneous activity in the BTHS mouse models may reflect a kind of anxiety-like behavior. *Taz*-KD mice in an open-field test demonstrated signs of elevated anxiety in conjunction with normal motor functions [52]. Notably, in the current study *Taz*-KO mice showed an age-related increase in locomotor activity during the light phase, when mice are typically inactive or asleep, potentially indicating increased anxiety and/or a decline in sleep quality. 

Since altered activity could not explain the difference in adiposity between *Taz*-KO mice and littermate controls, we performed indirect calorimetry studies to evaluate whether increased TEE may play a role. Normalized to total body weight, daily TEE was significantly higher in 12-month-old *Taz*-KO mice than that in age-matched littermates. However, differences at 3 and 6 months of age did not reach statistical significance (*p* = 0.07 and *p* = 0.08, respectively). Of important consideration, however, is the normalization factor used as a denominator in analyzing TEE. When corrected by body weight, TEE may be underestimated in obese mice, because a greater relative proportion of body weight is composed of white adipose tissue, which is metabolically less active than other lean tissues and organs [59,60]. Since body weights and adiposity differed so substantially between *Taz*-KO mice and *Wt* littermates, it is likely that these differences were an important factor in the calculated differences in TEE between *Wt* and *Taz*-KO mice, and may have played a quantitatively larger role in older mice where differences in adiposity were exaggerated. 

Patients with BTHS were found to have similar resting energy expenditure to controls, when energy expenditure was normalized to lean body mass [43]. However, it should be noted that attenuation of fat accumulation is a consistent feature of TAZ deficiency in mice. The protection against obesity in *Taz*-KO and *Taz*-KD mice, even when challenged with a high-fat diet [54], underscores the presence of a negative energy balance related to increases in energy expenditure and, likely, basal metabolic rate. Considering that mitochondrial dysfunction is central to the etiology of BTHS, it is likely that decreased efficiency of mitochondrial oxidative phosphorylation due to increased uncoupling of mitochondrial oxygen consumption from ATP production is compensated by increased substrate metabolism to generate the same amount of ATP [61]. At the whole-body level, this would manifest as higher levels of oxygen consumption, carbon dioxide production, and thus energy expenditure, as was observed in our study. 

In agreement, cardiomyocytes derived from induced pluripotent stem cells from human patients with BTHS have been shown to have increased mitochondrial proton leak across the inner membrane, resulting in elevated basal oxygen consumption [62]. Mitochondrial inefficiency is linked to higher rates of whole-body oxygen consumption in mice [61,63]. Alternatively, *Taz*-KD mouse studies have reported elevated mitochondrial content due to compensatory biogenesis [13,15,19], which can also contribute to higher rates of whole-body energy expenditure. 

Loss of TAZ affects whole-body substrate utilization, with studies on human patients suggesting that fatty acid oxidation is impaired, but partially compensated by increased glucose turnover [44,64]. In the current study, *Taz*-KO mice seem to recapitulate this increased preference for glucose as a fuel source for energy production, since they exhibited higher RER values at the ages of 6 and 12 months, reflecting greater oxidation of carbohydrates over fatty acids compared with *Wt* mice. It is likely that these abnormalities in substrate metabolism would become exacerbated with exercise, although this was not tested directly in the current study. In individuals with BTHS, whole-body fatty acid oxidization reportedly does not differ from controls at rest [43], yet submaximal exercise conditions revealed that the ability to upregulate fatty acid oxidation is severely blunted [44].

A leaner phenotype, coupled with improved use of glucose, is expected to confer protection against age-related deteriorations of glycemic control. Indeed, as the *Wt* mice aged, their glucose tolerance diminished, presumably due to progressive increases in adiposity, while the glucose tolerance of *Taz*-KO mice remained unchanged. During a GTT, the disposal of a glucose load is determined by insulin secretion, insulin action, and the effectiveness of glucose use [65]. Previously, Cole et al. (2016) reported that *Taz*-KD mice showed ~25% lower fasting plasma insulin levels at all timepoints during GTT with consequent hyperglycemia [54]. Cole et al. (2021) recently elucidated the cause of this hyperglycemia, identifying impaired insulin secretion during basal conditions (i.e., unstimulated low-glucose conditions) in *Taz*-KD mice, despite equivalent fold-increases in glucose-stimulated insulin secretion [66]. In the current study, plasma insulin levels and glucose utilization were not measured, so it is not possible to conclude the source of impaired glucose tolerance in the *Wt* mice, or attenuated deterioration in the *Taz*-KO mice. However, it is notable that in contrast to reports from the *Taz*-KD mice, hyperglycemia was not evident in *Taz*-KO mice, and in the youngest mice blood glucose measures from both genotypes were largely overlapping during the GTT. Thus, important differences between the *Taz*-KD and *Taz*-KO models exist and should be considered in translational work. 

Measurements of ITT did not indicate enhanced insulin sensitivity in the *Taz*-KO mice, although the ITT is more broadly designed for detecting insulin resistance rather than sensitivity [67]. In that regard, *Taz*-KO mice did not exhibit insulin resistance or age-related changes in overall insulin responsiveness, while the response to insulin gradually declined with increasing age in *Wt* mice, so that by 12 months of age, they appeared insulin resistant in comparison to their *Taz*-KO littermates.

The exercise capacity of *Taz*-KO mice was significantly attenuated relative to *Wt* littermates at each age analyzed. To the best of our knowledge, this study was the first to evaluate progressive changes in exercise capacity in the *Taz*-KO model. Notably, while an age-related decline was apparent in both genotypes, it occurred much earlier in *Taz*-KO mice. This decline was not associated with changes in skeletal muscles masses, which remained similar in size from 3 to 12 months of age within genotype groups. Additionally, this decline was unlikely to be solely related to changes in cardiac function, since it is reportedly relatively stable in adult BTHS patients [5,41,42] and mature *Taz*-cKO mice [23]. *Taz*-KO mice in the current study also did not experience any age-related change in heart weight. Due to the pleiotropic effects of cardiolipin depletion, it seems most likely that the exercise intolerance is multifactorial in origin. Lower cardiac contractile reserve, diminished skeletal muscle oxygen uptake [4], and impaired fuel substrate utilization [44] are all potential contributing factors that have been demonstrated to be present in BTHS patients. As these pathophysiological mechanisms are only partially understood, future investigations should focus on how these factors evolve over the lifespan in BTHS. In this regard, the *Taz*-KO mouse model presents an excellent opportunity to study the natural progression of this disease.

## 5. Conclusions

This study provides important information on the phenotype of the *Taz*-KO mouse model at three different ages, spanning from times corresponding to young adult to late middle-age in humans. *Taz*-KO mice have elevated TEE, which is likely driven by a higher basal metabolic rate resulting from mitochondrial inefficiencies, although further molecular and cellular studies on these parameters using this model system are needed to confirm this. The increased TEE apparently counteracts and supersedes the sufficient food intake observed in the mice, and thus is a likely mediator of the low adiposity in the *Taz*-KO mouse model. *Taz*-KO mice appear to be protected from age-related pathologies such as glucose intolerance and insulin resistance, and the lean phenotype of these animals and altered energetic substrate preferences are likely factors in this protection. However, these metabolic changes are also expected to contribute to compromised exercise capacity. While an elevated basal metabolic rate and increased substrate oxidation may offer protection against weight gain, impaired mitochondrial oxidative phosphorylation capacity will induce an energy deficit in times of increased energy demand, such as exercise. Data from *Taz*-KO mice support a theoretical model where mitochondrial energy production is insufficient to properly sustain the working heart and skeletal muscles, compromising exercise capacity.

Overall, this study found commonalities between *Taz*-KO mice and the human BTHS condition, supporting the use of this mouse model as a new resource for investigations into the pathophysiology and treatment of BTHS.

## Figures and Tables

**Table 1:** *Taz*-KO mice numbers at the time of sexing are below the predicted Mendelian ratio.

Sex	Genotype	Expected Number of Mice	Observed Number of Mice
Male	*Wt* (*Taz*^+/Y^)	176.75 (25%)	255 (36%)
	*Taz*-KO (*Taz*^Δ/Y^)	176.75 (25%)	43 (6%)
Female	*Wt* (*Taz*^+/+^)	176.75 (25%)	212 (30%)
	*Het* (*Taz*^Δ/+^)	176.75 (25%)	197 (28%)

Genotypic ratio of offspring at sexing resulting from *Wt* (*Taz*^+/Y^) male and heterozygous (*Taz*^Δ/+^) female breeding. The expected number of mice was calculated according to the total number of mice sexed, and was based on the expected Mendelian 1:1:1:1 ratio. Chi-squared test analysis indicated that discrepancies from the expected Mendelian ratios are statistically significant (*p* < 0.0001).

## Data Availability

Data are available upon reasonable request to the authors.
